# A novel aluminum foam structure for combined excellent wave attenuation and ventilation performance

**DOI:** 10.1038/s41598-025-91556-1

**Published:** 2025-03-01

**Authors:** Tiancong Hao, Xiaoning Yang, Aming Xie, Shuxin Deng, Bingbing Yu, Qingya Sun

**Affiliations:** 1 School of Safety Science and Engineering, Nanjing University of Science & Technology, Nanjing, 210094 China; 2 School of Mechanical Engineering, Nanjing University of Science & Technology, Nanjing, 210094 China

**Keywords:** Aluminum foam structure, explosive shock wave Attenuation mechanism, Crushing behavior, Ventilation resistance, Structural materials, Engineering

## Abstract

In this paper, a novel aluminum foam structure with wave attenuation and ventilation performance suitable for underground space is designed and prepared. It focuses on dynamic response of aluminum foam structure under explosion impact load and ventilation resistance at different wind speeds. Failure modes of each component are analyzed and attenuation mechanism of explosive shock wave are revealed. The results indicate that: under the synergistic action of the crushing behavior of the aluminum foam cells, the rough wall structure of the ventilation holes and the special diagonal square honeycomb-shaped structure, the wave attenuation effect of the aluminum foam structure is significantly improved. The max wave attenuation rate can reach to 99.1%. For this aluminum foam structure, the wind speeds are 4.32 m/s and 9.31 m/s while the ventilation resistances are 119.46 Pa and 641.44 Pa. It indicates its excellent ventilation performance. Therefore, the novel aluminum foam structure has a good application prospect in underground space construction.

## Introduction

In modern urban plan and design, the application of underground space has gradually become an important way to solve urbanization problems^1–3^. However, when an explosion occurs in an underground space building, the flow field of the blast shock wave is far more complex than the free field^4^, which poses a great threat to the safety of facilities and personnel in the underground protective structure^5,6^. At the same time, underground spaces usually lack natural ventilation, which leads to problems such as bad air quality and increased temperature, seriously affecting the comfort and safety of personnel and the normal function of equipment inside underground spaces^7,8^. Therefore, seeking a new structure that meets the demand for shockwave dissipation and the existence of ventilation performance is crucial for the development of underground spaces^9,10^.

After research, it is found that the way to attenuate the explosion shock wave in underground space structure is usually by blocking the propagation path with gravel and reinforced concrete wave dissipation structure^11–13^, but this way is not only difficult to address to the unexpected situation, but also difficult to take into account the needs of wave dissipation and ventilation. Therefore, seeking the application of new materials and structures has been a hot research topic.

Aluminum foam combines the intrinsic strength of metallic materials with the lightweight properties of foam materials^14–16^. It is a metal material characterized by low density, high specific strength, high stiffness and high vibration damping ability, showcasing outstanding energy absorption performance^17–20^, which makes it highly regarded in the fields of vehicle protection, engineering protection, and aerospace^21–23^. Numerous studies have shown that aluminum foam plays a great role in shock wave energy absorption. Li et al.^24^ studied the deformation and failure mode of aluminum foam sandwich board under air-blast loading by combining experiment and simulation. The results show that aluminum foam is the main energy-absorbing part in sandwich structure. Lu et al.^25^ studied dynamical response mechanism of a cladding sandwich panel with aluminum foam-filled tubular cores under impact load, and found that aluminum foam filler dissipated most of the impact energy. Liu et al.^26^ used wire mesh and aluminum foam to enhance the anti-knock properties of high-performance polymer composite walls. Compared with the traditional reinforced concrete wall, it can be shown that the new composite wall has better mechanical properties and blast absorption properties. Xu et al.^27^ explored the protective effect of closed cell aluminum foam layer on concrete column under low speed impact load by drop-weight test, and proved that aluminum foam layer can effectively absorb a large amount of impact energy, thus significantly reducing the impact kinetic energy. Therefore, the innovative structure based on aluminum foam material may provide a new technical route for underground space protection construction^28^. Li et al.^29^ applied closed-cell aluminum foam as a damping layer in tunnels, achieving excellent results. Juho^30^ et al. demonstrated through numerical simulations that aluminum foam can effectively absorb explosive energy in mine explosions. However, when applied in underground spaces, the unique characteristics of such environments must be considered, requiring a balance between explosion resistance and ventilation performance. This critical aspect is not adequately addressed in the existing literature.

In this study, a unique multilayer aluminum foam structure is designed, it balances both wave dissipation performance and ventilation requirements. The aim is to explore the potential application of aluminum foam for attenuating explosion shock waves in underground spaces, and to provide new ideas for safety and ventilation effects in underground spaces.

## Experimental investigation

### Sample structure design

**Fig. 1:**
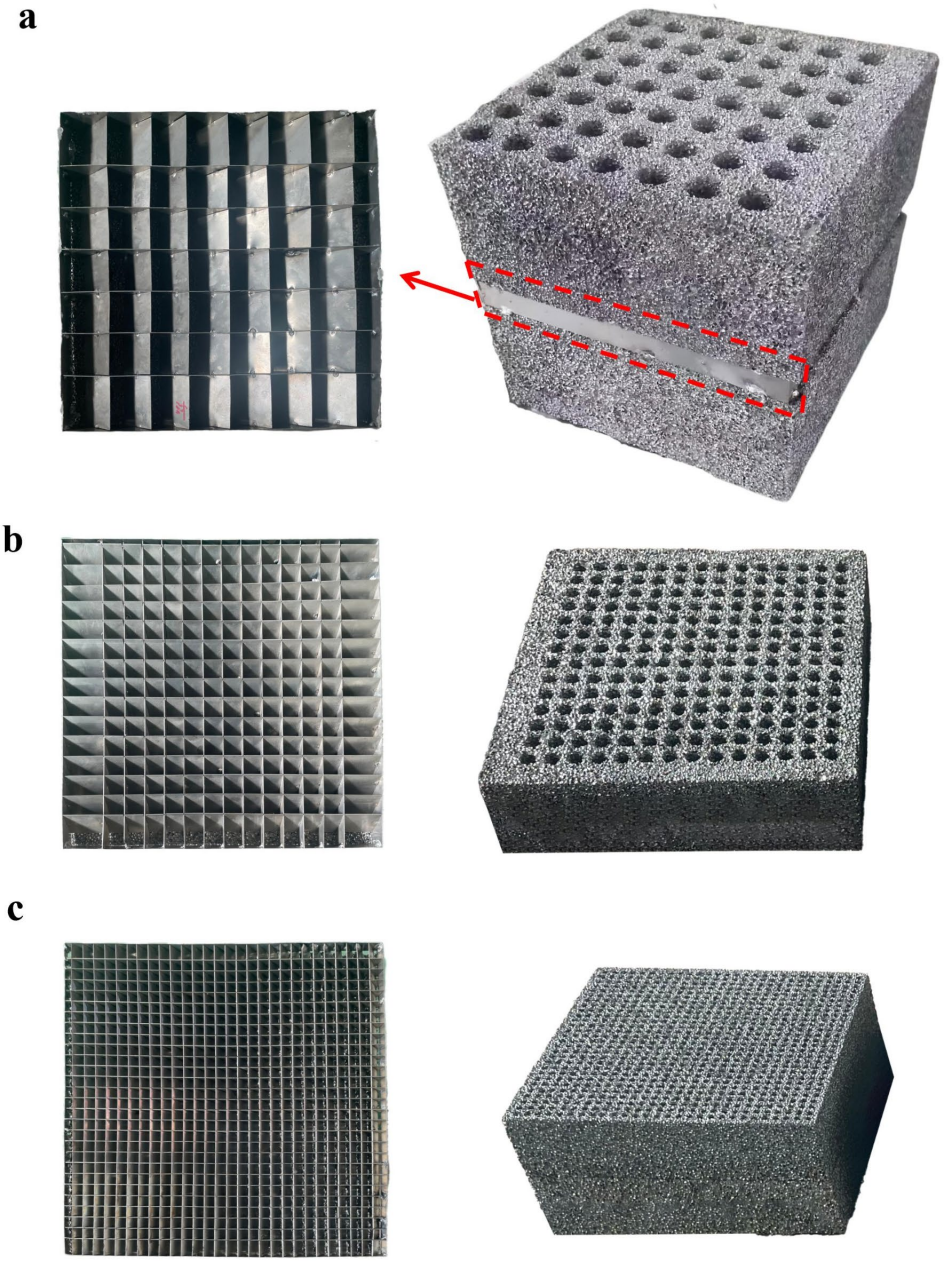
The sample of aluminum foam shock wave dissipation structure and diagonal square honeycomb-shaped aluminum plate: **a** 7 × 7; **b** 14 × 14; **c** 28 × 28

The design of closed-cell aluminum foam shock wave dissipation structure is presented in Fig. 1. It can be seen that the entire configuration comprises three distinct layers: the upper layer features closed-cell aluminum foam, the middle layer incorporates a diagonal square honeycomb-shaped aluminum plate and the lower layer consists of closed-cell aluminum foam. To ensure efficient ventilation throughout the air exchange process, systematic ventilation holes are pre-fabricated within the closed-cell aluminum foam. Specifically, to prevent the air shock wave from propagating linearly through the ventilation holes of the wave attenuation structure during an explosion, the ventilation holes in the upper and lower layers are arranged in a dislocated pattern. Additionally, the middle layer structure is inclined at a 45° angle to connect the ventilation holes of the upper and lower layers. To ensure adequate ventilation performance, the height of the middle layer structure is determined by the size of the ventilation holes. The middle layer structure varies according to the number and arrangement of ventilation holes in the aluminum foam, as shown in Fig. 1a, b and c.

The porosity of the aluminum foam utilized in this study is 65% and it is composed of pure aluminum materials. Both the upper and lower aluminum foam sections share the same dimensions, measuring 500 mm × 500 mm × 220 mm. The diagonal square honeycomb-shaped aluminum plate is constructed from pure aluminum materials, featuring a tilt angle of 45°. The research focuses on 3 sets of experiments designed to investigate the impact of shock wave pressure, ventilation hole size and arrangement of ventilation hole distribution on the wave attenuation performance of the aluminum foam structure. A comprehensive overview of the test conditions is provided in Table 1, it can be seen that the diameter of the first sample is 20 mm, the holes arrangement is 14 × 14, the thickness of core structure is made as 30 mm, and the mass of explosion source is 0.2 Kg, which is defined as AF14-1. The second sample, labeled AF14-2, shares the same specifications except for an increased explosion source mass of 0.6 Kg. The third sample, designated as AF07, has the holes of 40 mm diameter, a 7 × 7 holes arrangement, a core structure thickness of 60 mm and an explosion source mass of 0.6 Kg. The diameter of the fourth sample is 15 mm, it features a 28 × 28 holes arrangement, a core structure thickness of 30 mm and an explosion source mass of 0.6 Kg, which is identified as AF28.

**Table 1:** Test conditions

Sample	Aluminum Foam Thickness (mm)	TNT/kg	Explosive source location/m	Hole diameter (mm)	Thickness of core structure/mm	Hole arrangement
AF14-1	220	0.2	1	20	30	14 × 14
AF14-2	220	0.6	1	20	30	14 × 14
AF07	220	0.6	1	40	60	7 × 7
AF28	220	0.6	1	15	25	28 × 28

### Explosion experiment setup

A novel experimental apparatus, featuring a reinforced concrete/steel cylinder composite structure, has been designed. The reinforced concrete component forms the latter part of the apparatus, with internal dimensions of 0.5 m × 0.5 m × 0.8 m. During the experiment, the sample was placed inside this section. To fix the aluminum foam samples and prevent direct displacement due to shock waves, three steel reinforcements are strategically positioned 0.3 m from the tail of the reinforced concrete structure. A detachable square steel cylinder, with internal dimensions of 0.5 m×0.5 m×1 m, is affixed in front of the reinforced concrete structure using rivets. The explosion source is centrally located within the steel cylinder, facilitating the transmission of the air shock wave through the steel cylinder to the interior of the sample.

**Fig. 2:**
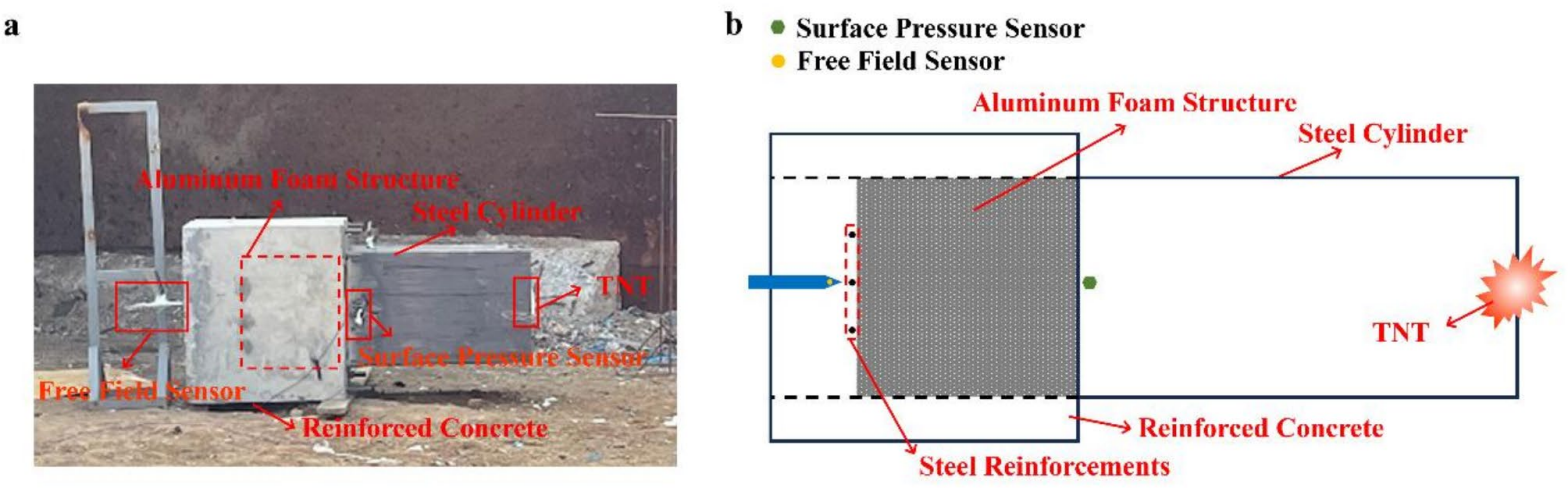
The setup of explosion experiment: **a** On-site experimental setup; **b** Schematic diagram of the experimental setup.

The explosive source used in this test is cylindrical TNT, characterized by a length-to-diameter ratio of 1:1. Electric detonators are utilized to detonate. A PCB surface pressure sensor (PCB/113B21) is strategically placed at the connection point between the reinforced concrete structure and the steel cylinder, positioned 0.25 m away from the upper surface of the steel cylinder. This sensor measures the incident pressure during the experiment. Additionally, a PCB free field sensor (PCB/137B24B) is situated at the center of the cross-section of the reinforced concrete structure, 0.2 m away from the back of the structure. This sensor is used to measure the residual pressure. The experimental setup is shown in Fig. 2, where Fig. 2a represents the on-site arrangement of the experiment, and Fig. 2b provides a schematic diagram of the experimental setup.

### Ventilation performance test

In the ventilation test, a variable power fan is used to provide the wind source. Wind speed measurements at the inlet and outlet of the reinforced concrete/steel cylinder composite structure are conducted using a handheld anemometer (Testo 510), while a hand-held differential pressure meter (testo 512) is used to measure the wind pressure at the same location. The measurement points for pressure and velocity encompass 10 equidistant locations both longitudinally and transversely, positioned both in front of and behind the aluminum foam structure. The final pressure and velocity values are derived from the average of these measurements, ensuring a comprehensive representation of the airflow characteristics.

## Results and discussion

### Characteristic of blast loading

**Fig. 3:**
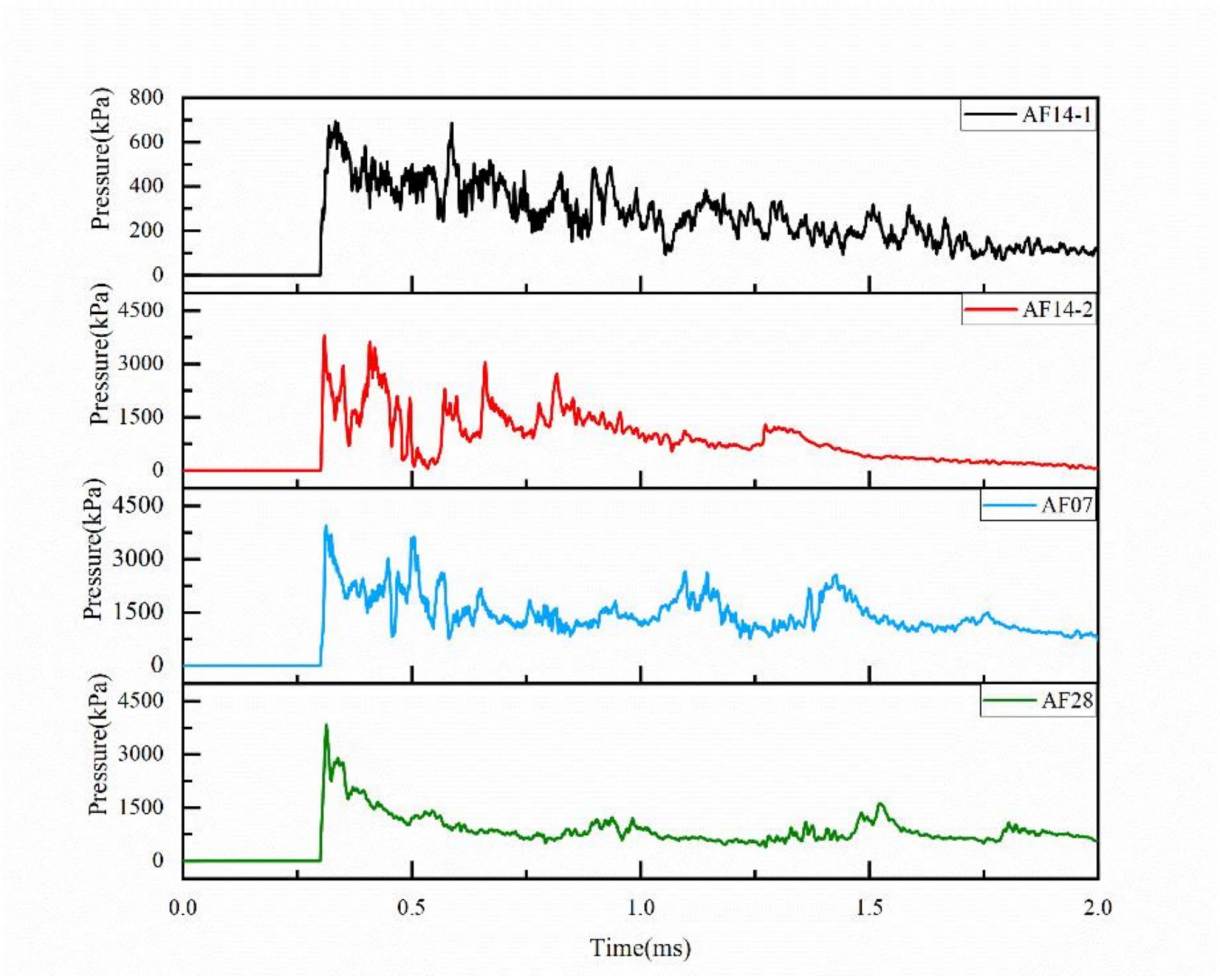
The incident shock wave pressure of AF14-1, AF14-2, AF07 and AF28.

Figure 3 showed the pressure-time curves of incident shock wave of aluminum foam wave dissipation structure. It can be seen that the incident shock wave pressure of AF14-1 was less, and the peak pressures was 0.693 MPa. The peak pressure of the incident shock wave of AF14-2, AF07 and AF28 were similar and higher than AF14-1,which were 3.793 MPa, 3.943 MPa and 3.833 MPa, respectively. By comparing the four groups of pressure curves, it can be found that the pressure change trends of explosion shock wave were basically the same, and multiple peaks still appeared after the maximum pressure value, which was caused by the reflection phenomenon of shock wave on the wall.

**Fig. 4:**
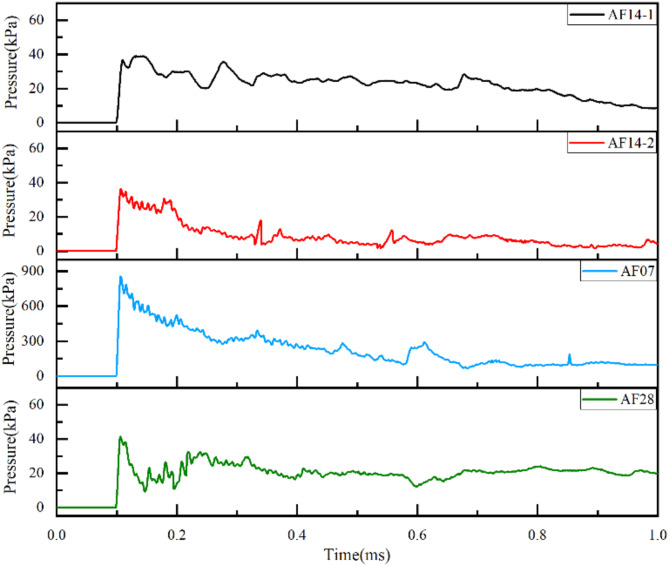
The residual shock wave pressure of AF14-1, AF14-2, AF07 and AF28.

The residual pressure-time curves of the explosion shock wave after passing through the aluminum foam wave dissipation structure were showed in Fig. 4. When the explosion shock wave was transmitted to the back of the aluminum foam structure, the peak pressure values of the four groups of samples decreased greatly at the same time. It was found that the maximum peak residual pressure values of AF14-1, AF14-2, AF07 and AF28 were 0.039 MPa, 0.036 MPa, 0.853 MPa and 0.041 MPa by measurement, which the corresponding wave dissipation rate were 94.4%, 99.1%, 78.4% and 98.9%, respectively. All the aluminum foam wave dissipation structures showed excellent attenuation properties of explosion shock wave under different conditions. Comparing AF14-1 and AF14-2, it can be found that when the aluminum foam structure is the same, the higher the shock wave pressure is, the better the wave dissipation effect is. According to pressure-time curves of AF14-2,AF07 and AF28, the shock wave dissipation effect of aluminum foam structure increased firstly and then decreased with the increasing of the number of prefabricated ventilation holes.

### Failure behaviors

**Fig. 5:**
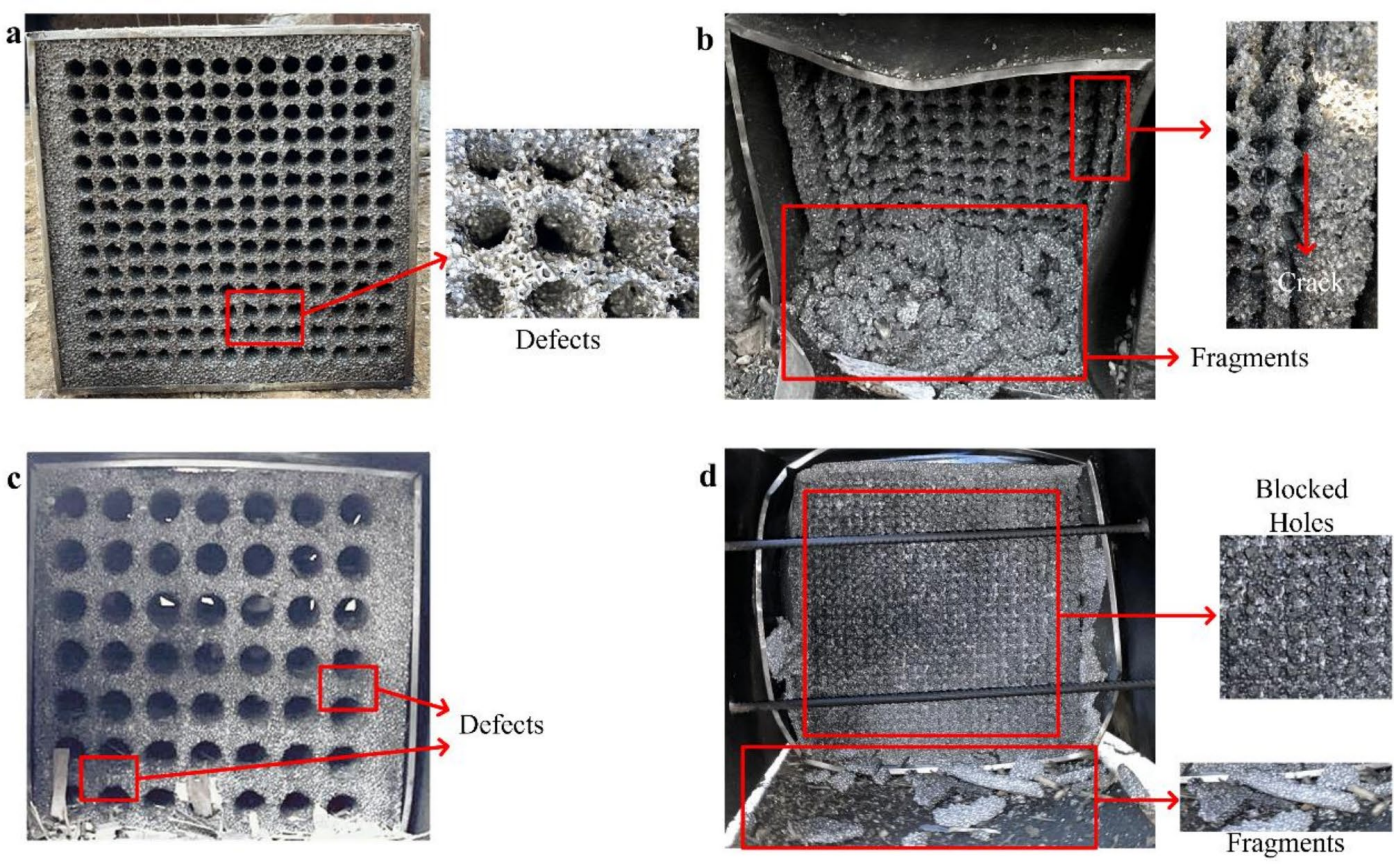
The morphology of the aluminum foam structure on the blast-facing surface: **a** AF14-1; **b** AF14-2; **c** AF07; **d** AF28.

The morphology of the aluminum foam structure on the blast-facing surface was depicted in Fig. 5 after the explosive impact. Only a few minor defects appeared in the connecting wall between the ventilation holes in AF14-1, and its crushing deformation was small (Fig. 5a). It was because the incident shock wave pressure of AF14-1 was low, and most of the explosion shock wave was reflected and diffracted in the side wall of the ventilation hole of aluminum foam structure. Thus, the explosion shock wave was effectively attenuated, leading that AF14-1 was basically undamaged. When the pressure of the incident shock wave was increased from 0.693 MPa to 3.793 MPa, the damage of the AF14-2 became more serious, and the aluminum foam structure occurred obvious crushing phenomenon^31^. According to Fig. 5b, it can be found that a large number of fragments accumulated in the front of the structure with the cracks occurring connection wall of the outermost ventilation holes, and a deep curved pit was formed outward from the center of the AF14-2. The kinetic energy generated by the explosion shock wave was effectively reduced by deformation and crushing of aluminum foam, so as to achieve the ideal wave attenuation effect. In Fig. 5c, the structural surface of AF07 did not change significantly with the increase of the diameter of the ventilation holes, resulting in greater residual pressure. Figure 5d showed the morphology of AF28 on the blast-facing surface. As the increase of the number of ventilation holes and the decrease of the size of ventilation holes, the structural strength of AF28was reduced, which led to fault phenomenon of the upper layer aluminum foam and the complete block of the ventilation holes after the AF28 was squeezed and deformed by shock wave.

**Fig. 6:**
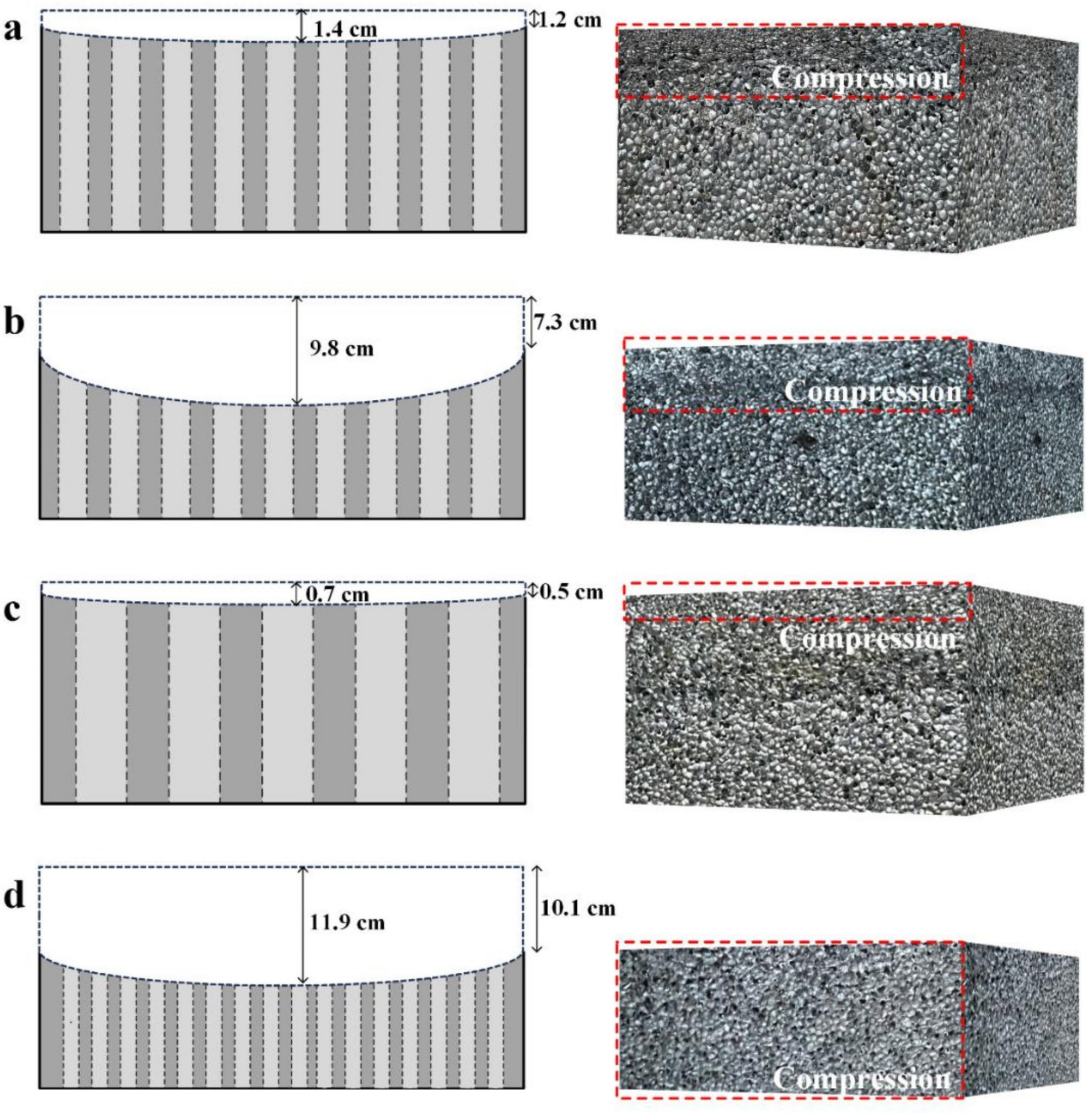
Side morphology of aluminum foam structure on the blast-facing surface: **a** AF14-1; **b** AF14-2; **c** AF07; **d** AF28.

Figure 6 showed the side morphology of aluminum foam structure on the blast-facing surface, which the left part was diagram of depression degree of structure and the right part was diagram of structural height and the change of cell size on the outer surface. In Fig. 6a, the compression distance of AF14-1 was 1.2 cm at the edge and 1.4 cm at the center. It can be seen that the cells sizes of AF14-1 became smaller after the explosion impact, which proved that the aluminum foam structure had obvious crushing behavior. By comparing AF14-1, it can be found that the deformation degree of AF14-2 greatly increased with the increase of incident shock pressure, which the compression distance was 7.3 cm at the edge and 9.8 cm at the center (Fig. 6b). In Fig. 6c, the deformation degree of AF07 was relatively low, the compression distance was only 0.5 cm at the edge and 0.7 cm at the center. It was due to as the diameter of the ventilation holes increased, the shock waves could pass through the structure with less attenuation. According to Fig. 6d, It can be seen the compression distance of AF28 was 10.1 cm at the edge and 11.9 cm at the center.

**Fig. 7:**
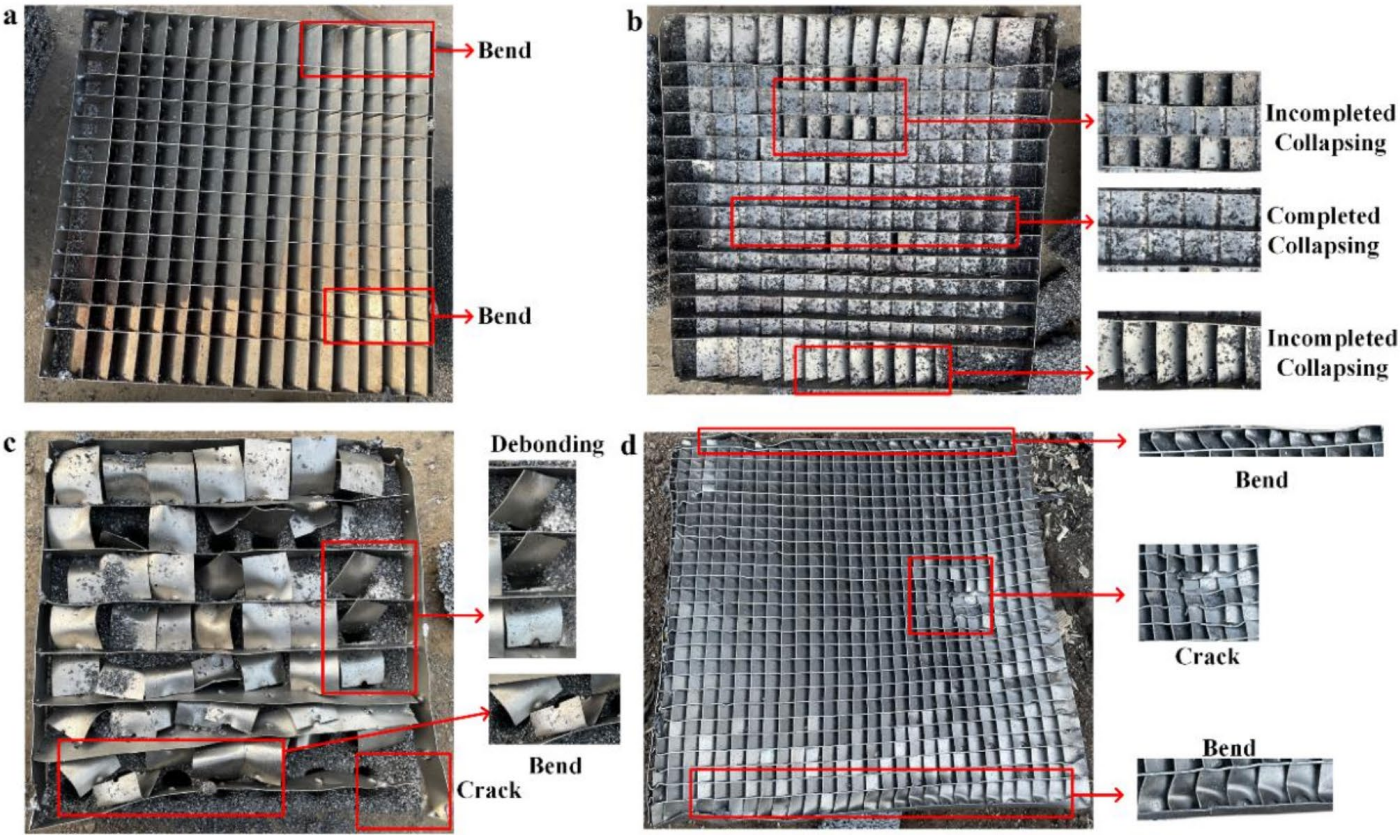
The morphology of diagonal square honeycomb-shaped structure: **a** AF14-1; **b** AF14-2; **c** AF07; **d** AF28.

The morphology of diagonal square honeycomb-shaped structure was showed in Fig. 7. Only 7% of the aluminum sheets of AF14-1 were bent with low incident shock wave pressure (Fig. 7a). In Figs. 7b and 17% of the aluminum sheets of AF14-2 had incomplete collapse, and the remaining 83% of the aluminum sheets had complete collapse at 180°. The collapse of the aluminum sheets can completely cover the lower ventilation holes of the aluminum foam structure, thus blocking the transmission of the explosion shock wave. The diagonal square honeycomb-shaped structure of AF07 was seriously damaged by the explosion shock wave. All of the aluminum sheets had been fallen off from the frame with frame suffered obvious fracture, resulting in the complete failure of the shock wave dissipation performance of AF07 (Fig. 7c). In Figs. 7d and 82% of the aluminum sheets of AF28 had incomplete collapse, and the remaining 18% of the aluminum sheets had complete collapse. Under the action of shock wave, aluminum sheets around the outside of diagonal square honeycomb-shaped structure were squeezed and bent.

**Fig. 8:**
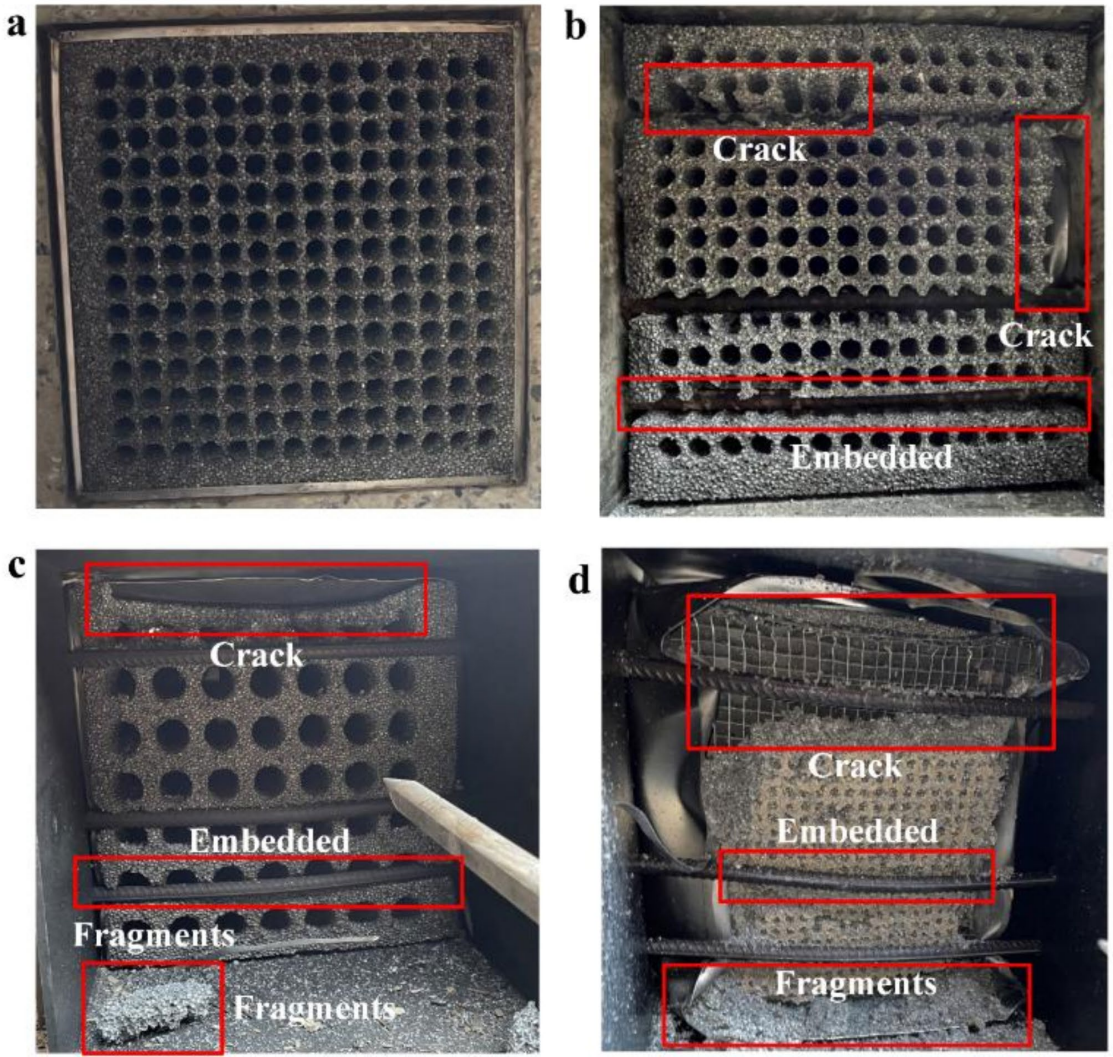
morphology of aluminum foam structure on the back blast surface: **a** AF14-1; **b** AF14-2; **c** AF07; **d** AF28.

Figure 8 depicted the backblast surface of the shock wave dissipation structure. Because of the low incident pressure, there was no significant change on the backblast surface of AF14-1(Fig. 8a). As the incident pressure increased, AF14-2(Fig. 8b), AF07(Fig. 8c) and AF28(Fig. 8d) showed situations where the steel reinforcements were embedded into the aluminum foam structure. This was because of the immense thrust generated by the explosive shock wave. Measurements indicated that the steel reinforcement was embedded 5.6 cm into AF14-2, 2.3 cm into AF07. For AF28, the strength of the aluminum foam on the backblast surface completely degraded as a result of increased ventilation area and increased number of ventilation holes, resulting in complete damage to the backblast surface.

### The result of ventilation

**Fig. 9:**
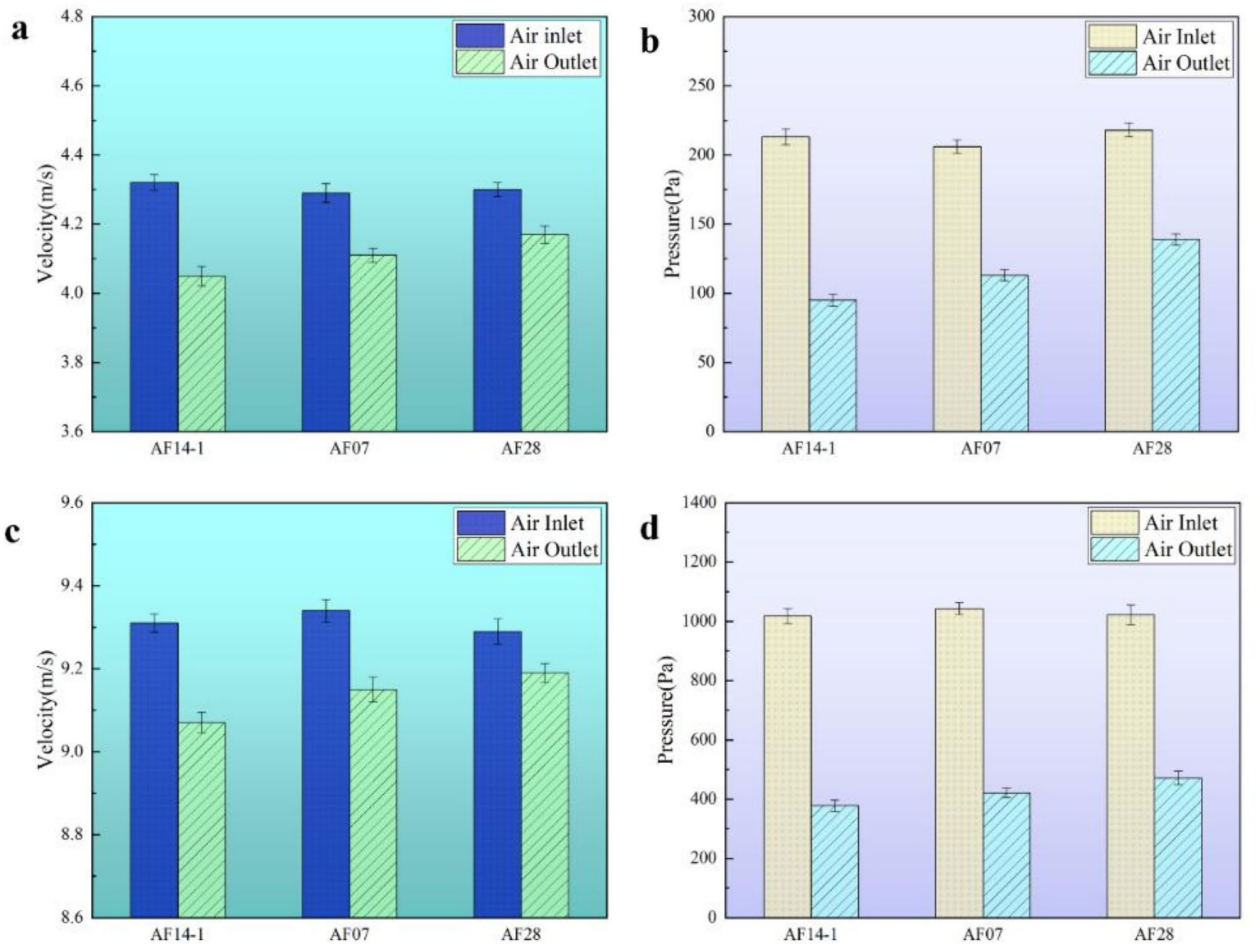
The ventilation velocities and pressures: **a** wind speeds at the power of 1KW; **b** wind pressures at the power of 1KW; **c** wind speeds at the power of 2.2KW **a** wind pressures at the power of 2.2KW.

The ventilation velocities and pressures for AF14-1, AF07 and AF28 at two fan power levels were illustrated in Fig. 9. Based on the airflow velocity and pressure readings, the ventilation resistance of the aluminum foam structure could be determined by the airflow energy equation (Eq. 1).

1 $$\:{p}_{1}+\frac{\rho\:}{2}{v}_{1}^{2}+\left({\gamma\:}_{a}-\gamma\:\right)={p}_{2}+\frac{\rho\:}{2}{v}_{2}^{2}+{p}_{1-2}$$

Where, $\:{p}_{1}$ is the pressure at the inlet, Pa; $\:{p}_{2}$ is the pressure at the outlet, Pa; $\:{\gamma\:}_{a}-\gamma\:$ is the atmospheric pressure difference between the location of the inlet and outlet, Pa; $\:\rho\:$ is the density of air, kg/m³; $\:{v}_{1}$ is the wind speed at the inlet, m/s; $\:{v}_{2}$ is the wind speed at the outlet, m/s.

The ventilation velocities and pressures for AF14-1, AF07 and AF28 at a fan power of 1 kW were shown in Fig. 9a and b. It was measured that the inlet velocity, inlet pressure, outlet velocity, and outlet pressure of the AF14-1 were 4.32 m/s, 213 Pa, 4.05 m/s and 95 Pa, respectively. The parameters for AF07 were as follows: the inlet airflow velocity was 4.29 m/s, the inlet pressure was 206 Pa, the output velocity was 4.11 m/s, and the outlet pressure was 113 Pa. Concerning AF28, the velocity at the inlet was 4.3 m/s, the pressure at the inlet was 218 Pa, the velocity at the outlet was 4.17 m/s, and the outlet pressure was 139 Pa. The ventilation resistances for AF14-1, AF07, and AF28 at a fan power of 1 KW could be calculate as 119.46 Pa, 93.96 Pa and 79.99 Pa, respectively.

As the fan power increased from 1 kW to 2.2 kW, the airflow velocities and pressures for AF14-1, AF07, and AF28 were displayed in Fig. 9c and d. Specifically, the measured values were as follows: At the AF14-1 inlet, the velocity was 9.31 m/s and the pressure was 1018 Pa, while at the AF14-1 outlet, velocity was 9.07 m/s, and the pressure was 378 Pa. For AF07, the inlet velocity was 9.34 m/s, the inlet pressure was 1043 Pa, the outlet velocity was 9.15 m/s, and the outlet pressure was 421 Pa. In the case of AF28, the inlet velocity was 9.29 m/s, the inlet pressure was 1022 Pa, the outlet velocity was 9.18 m/s, and the outlet pressure was 472 Pa. The calculated ventilation resistances for AF14-1, AF07 and AF28 at a fan power of 2.2 kW were 641.44 Pa, 623.14 Pa and 550.66 Pa, respectively.

It was evident that the ventilation effectiveness of the aluminum foam structure improved with either the ventilation area or the size of each ventilation hole increased. The ventilation performance of the aluminum foam structure designed in this study far exceeded the standard when compared to the AQ1028-2006 standard.

### Action mechanism of the aluminum foam structure

To further analyze the failure modes and wave attenuation mechanisms of aluminum foam structures under explosion shock waves, numerical simulations were conducted on sample AF14-2.

**Fig. 10:**
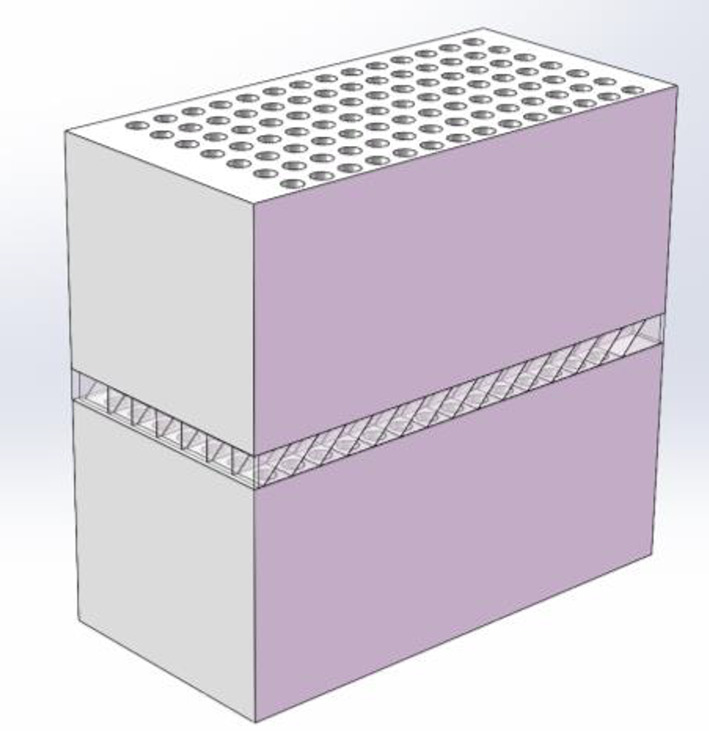
Half-geometry model of the aluminum foam structure.

As shown in Fig. 10, due to the symmetry of the structure, a half-model was created in SolidWorks and then imported into HyperMesh for mesh generation. Finally, finite element simulations were conducted using LS-DYNA. The MAT_CRUSHABLE_FOAM model was employed to simulate the aluminum foam material, while the MAT_POWER_LAW_PLASTICITY model was used for the diagonal square honeycomb-shaped aluminum plate.

**Fig. 11:**
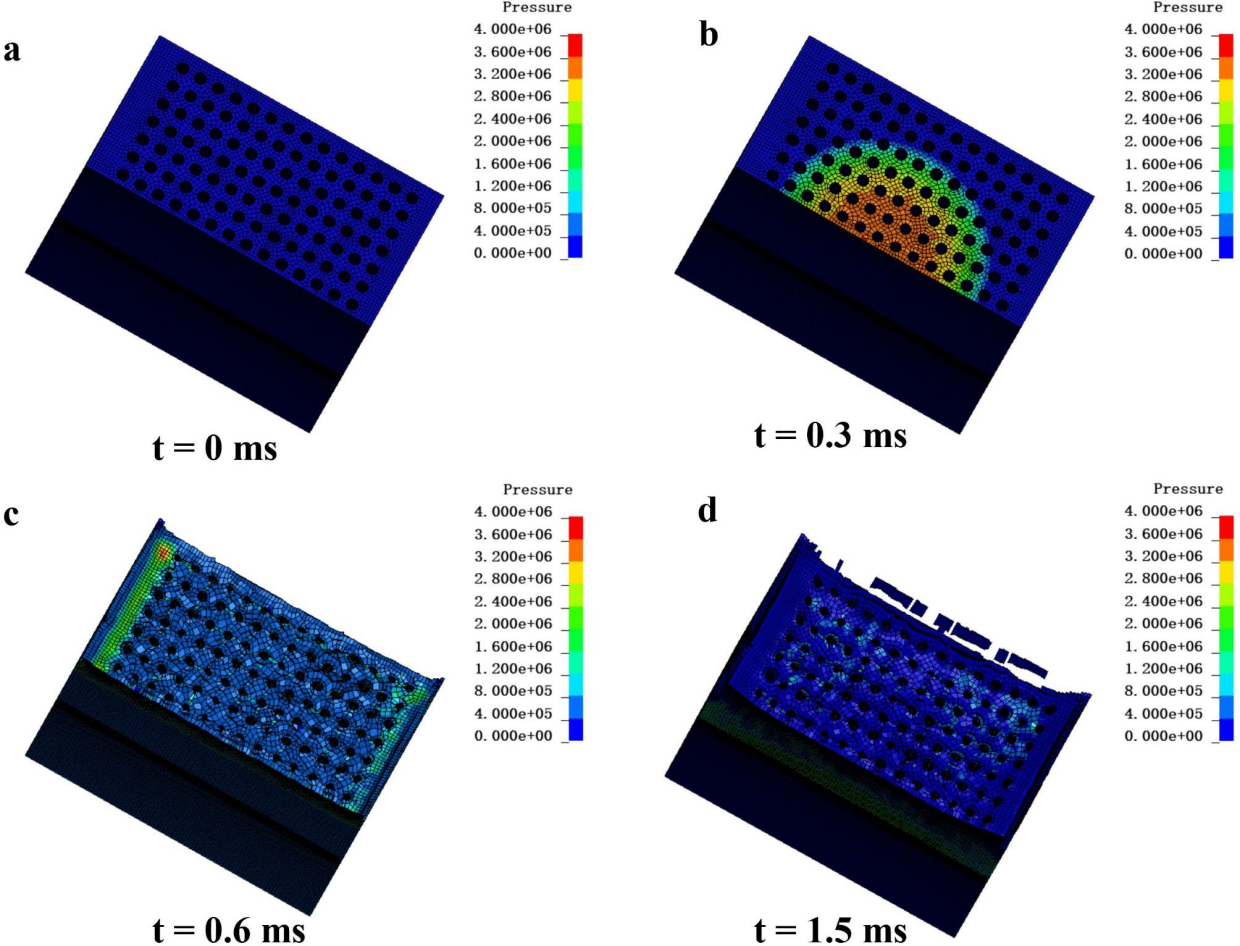
Pressure distribution cloud diagram: **a** t = 0 ms; **b** t = 0.3 ms; **c** t = 0.6 ms; **d** t = 1.5 ms.

The simulated visualization of the aluminum foam structure during the explosion shock wave process is shown in the pressure distribution cloud diagrams in Fig. 11. These diagrams provide a clear and intuitive depiction of the failure mode and pressure distribution caused by the shock wave. Figure 11a depicts the initial state of the overall model. Figure 11b illustrates the pressure distribution at t = 0.3 ms as the explosion shock wave reaches the blast-facing surface, where the pressure is significantly higher near the central region. This pressure concentration is the primary reason for the formation of a concave crater in the damaged upper aluminum foam. In Fig. 11c, the pressure distribution reveals that at t = 0.6 ms residual pressure remains on the blast-facing surface as the explosion shock wave propagates through it, particularly at the outermost ventilation holes. This residual pressure is the primary cause of cracks observed in the connecting walls of the ventilation holes. From the damage morphology, it can be seen that the aluminum foam has already been compressed by the explosion shock wave at this stage, preliminarily forming a concave crater. Figure 11d illustrates the damage state of the upper aluminum foam at t = 1.5 ms, showing a crater with a deep center and shallower edges, accompanied by the generation of debris; The pressure distribution cloud diagram reveals that as the shock wave propagates into the aluminum foam, its pressure is significantly reduced. This indicates that the aluminum foam effectively attenuates the energy of the explosion shock wave through compression deformation. The results from the numerical simulation closely align with those observed in experimental tests, confirming the accuracy of the simulation.

**Fig. 12:**
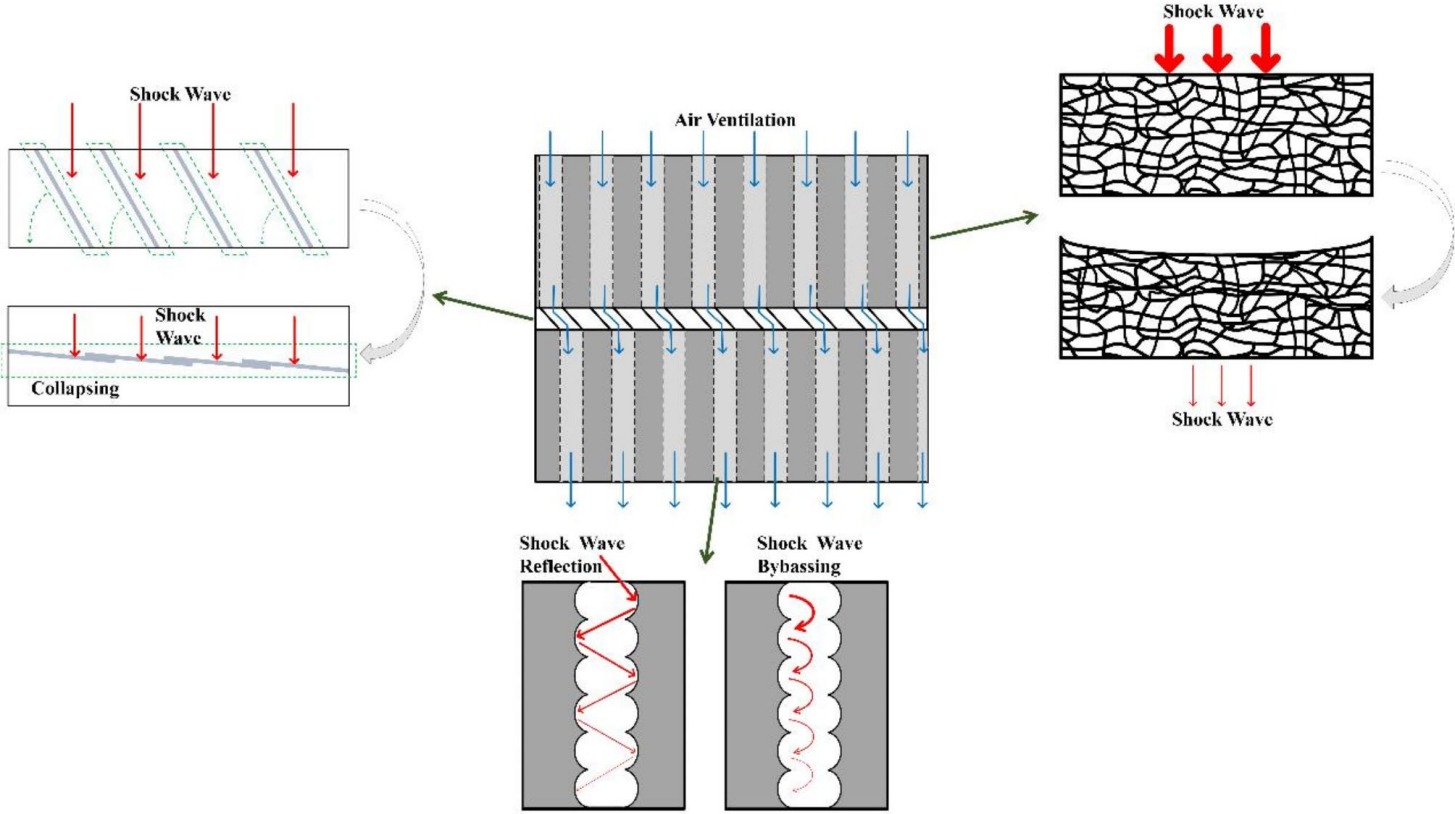
Attenuation mechanism of aluminum foam structure.

Attenuation mechanism of novel aluminum foam structure to explosion shock wave was showed in Fig. 12. As the explosion shock wave did not reach the surface of the aluminum foam structure, it could carry out normal air ventilation with excellent ventilation performance. When the explosion shock wave acts on the aluminum foam structure, it mainly carries on the wave attenuation in three ways. The first step, the internal cells of aluminum foam material are compressed with collapse deformation occurring. In this process, part of the shock wave is converted into kinetic energy and thus consumed. Next, the inner wall of the ventilation holes is very rough due to the existence of cellular pores. When the shock wave passes through the ventilation holes, it will be reflected and diffracted in the inner cell of aluminum foam, thus part of the shock wave energy is attenuated. The third step, after the shock wave is transmitted to the middle layer diagonal square honeycomb-shaped structure, the aluminum sheet will be collapsed by the shock wave and form a steel sheet-like structure on its own, and then the ventilation holes in the lower layer are completely blocked. This effectively blocks the further transmission of the shock wave.

## Conclusion

A novel wave attenuation and ventilation structure using aluminum foam material is proposed. Experimental investigations are conducted to study the wave attenuation and ventilation performance of the foam aluminum structure. The paper analyzes the wave attenuation and ventilation performance of the foam aluminum structure under different arrangements of ventilation holes. From the presented experimental results, we come to the following conclusion:

(1)The wave attenuation rate of AF14-1,AF14-2,AF07 and AF28 are 94.4%, 99.1%, 78.4% and 98.9%, respectively.

(2)At a fan power of 1 kW, the ventilation resistances for AF14-1, AF07 and AF28 are 119.46 Pa, 93.96 Pa and 79.99 Pa, respectively. When the fan power is increased to 2.2 kW, the ventilation resistances become 641.44 Pa, 623.14 Pa and 550.66 Pa for AF14-1, AF07, and AF28, respectively. The aluminum foam structure demonstrates excellent ventilation performance, significantly surpassing the AQ1028-2006 standard.

(3)The deformation of cells in aluminum foam, the collapse of diagonal square honeycomb-shaped structure and the irregular ventilation hole walls can absorb a significant amount of shock wave energy. This is considered the primary source of energy attenuation capability in aluminum foam structures.

## Data Availability

Data will be made available on request.The datasets used or analysed during the current study available from the corresponding author on reasonable request.All data generated or analysed during this study are included in this published article. The datasets used or analysed during the current study available from the corresponding author on reasonable request. All data generated or analysed during this study are included in this published article.

## Declarations

### Additional information

**Correspondence** and requests for materials should be addressed to Q.S or S.D.

### Reprints and permissions information

is available at www.nature.com/reprints.

**Publisher’s note** Springer Nature remains neutral with regard to jurisdictional claims in published maps and institutional affiliations.
